# Cnidarian Pattern Recognition Receptor Repertoires Reflect Both Phylogeny and Life History Traits

**DOI:** 10.3389/fimmu.2021.689463

**Published:** 2021-06-23

**Authors:** Madison A. Emery, Bradford A. Dimos, Laura D. Mydlarz

**Affiliations:** Department of Biology, University of Texas at Arlington, Arlington, TX, United States

**Keywords:** innate immunity, non-bilaterian, pattern recognition receptors, Toll-like receptor, NOD-like receptor, RIG-I like receptor, C-type lectin

## Abstract

Pattern recognition receptors (PRRs) are evolutionarily ancient and crucial components of innate immunity, recognizing danger-associated molecular patterns (DAMPs) and activating host defenses. Basal non-bilaterian animals such as cnidarians must rely solely on innate immunity to defend themselves from pathogens. By investigating cnidarian PRR repertoires we can gain insight into the evolution of innate immunity in these basal animals. Here we utilize the increasing amount of available genomic resources within Cnidaria to survey the PRR repertoires and downstream immune pathway completeness within 15 cnidarian species spanning two major cnidarian clades, Anthozoa and Medusozoa. Overall, we find that anthozoans possess prototypical PRRs, while medusozoans appear to lack these immune proteins. Additionally, anthozoans consistently had higher numbers of PRRs across all four classes relative to medusozoans, a trend largely driven by expansions in NOD-like receptors and C-type lectins. Symbiotic, sessile, and colonial cnidarians also have expanded PRR repertoires relative to their non-symbiotic, mobile, and solitary counterparts. Interestingly, cnidarians seem to lack key components of mammalian innate immune pathways, though similar to PRR numbers, anthozoans possess more complete immune pathways than medusozoans. Together, our data indicate that anthozoans have greater immune specificity than medusozoans, which we hypothesize to be due to life history traits common within Anthozoa. Overall, this investigation reveals important insights into the evolution of innate immune proteins within these basal animals.

## Introduction

Animals sense and interact with microbes through their immune system (1). The majority of knowledge about immune system function stems from studies in vertebrates, which interact with microbes through both an innate and an adaptive immune system (2, 3). In contrast, invertebrates rely on innate immunity to detect and respond to microbes. Pattern recognition receptors (PRRs) are key components of innate immunity that detect both danger associated molecular patterns (DAMPs) and microbe associated molecular patterns (MAMPs), activating downstream signaling pathways following this recognition (4). PRRs are drivers of immune specificity in invertebrates, as diverse repertoires of receptors are needed in order to generate a microbe specific immune response (5).

The four best studied families of PRRs are Toll-like receptors (TLRs), retinoic acid-inducible gene I-like receptors (RLRs), nucleotide-binding oligomerization domain-like receptors (NLRs), and C-type lectins (CTLs) (6, 7). While it was originally thought that many of these PRR families first arose in vertebrates, studies following the advent of next generation sequencing revealed that TLRs, RLRs, NLRs, and CTLs are present in basal metazoans and thus are evolutionarily ancient (8–12). Studying PRRs in basal taxa informs our understanding of PRR evolution and more broadly immune evolution.

Cnidaria is a basal phylum sister to Bilateria with over 10,000 species spanning three major clades: Anthozoa, Medusozoa, and Endocnidozoa which span a diverse array of life history strategies ranging from sedentary to planktonic to parasitic (13). Despite their primitive morphology, cnidarian genomes are complex and contain a repertoire of innate immune genes unexpectedly similar to mammals (14, 15). Due to this, cnidarians are exceptional candidates for investigations into innate immune evolution as they are not only basal, but also have immune repertoires are not reduced like the nematode model *Caenorhabditis elegans* or derived in function like the arthropod model *Drosophila melanogaster* (15–17). Additionally, common life history traits within Cnidaria have been linked to immunity. Several cnidarian taxa in Anthozoa and Medusozoa form intracellular nutritional symbiosis with algae in the family Symbiodiniaceae, the maintenance of which has been linked to several different PRRs as well as NFκB signaling (18). Similarly, many cnidarians have colonial body plans, and likely have a larger need for allorecognition and thus immune specificity (13, 19, 20). Therefore, the wide variety of life history traits found within cnidaria has the potential to create a gradient of selective pressures for immune specificity, which likely is reflected in their PRR repertoires.

Toll-like receptors (TLRs) are transmembrane PRRs that are capable of recognizing a wide variety of ligands including bacterial cell wall components, viral RNA, and developmental cues (21–23). Canonically, TLRs consist of extracellular leucine rich repeats (LRRs) which bind to DAMPs and MAMPs and an intracellular Toll/interleukin-1 receptor (TIR) domain that activates signal transduction through protein-protein interactions with other TIR domain-containing proteins (21, 22, 24). Several pathways can be activated by TLRs following ligand engagement, including the MAPK, IFN, and NFκB pathways (22, 24). Within Cnidaria, prototypical TLRs have been identified in several anthozoan species, and functional studies indicate that the *Nematostella vectensis* TLR functions in pathogen recognition, activation of NFκB signaling, and development (25, 26). Additionally, a medusozoan species lacking prototypical TLRs, *Hydra vulgaris*, is still capable of TLR-NFκB signaling through TLR-like proteins containing a transmembrane domain and an intracellular TIR domain that appear to interact with transmembrane proteins with extracellular leucine rich repeats to perform the function of the prototypical TLR (27).

Retinoic acid-inducible gene I-like receptors (RLRs) are cytosolic PRRs that detect intracellular viral RNA (28–30). Mammals have three RLRs: RIG-I, MDA5, and LGP2. All RLRs have a central ATPase containing DExD/H box helicase domain and a C-terminal regulatory domain (28, 30). RIG-I and MDA5 also contain CARD domains which in mammals interact with the CARD domain of signaling adaptor MAVS to initiate downstream signaling, activating transcription factors IRF3 and NFκB and ultimately resulting in an antiviral response (31, 32). Additionally, RIG-I and LGP2 have a repressor domain (RD) within the C-terminal regulatory domain (28, 30). As LGP2 lacks CARD domains, it is unable to initiate antiviral signaling and instead likely acts as a concentration dependent biphasic switch in mammals, positively regulating MDA5 at low concentrations and negatively regulating RIG-I and MDA5 at high concentrations (33–35). Some RLRs with sequences most similar to RIG-I have been identified in the *N. vectensis* genome (9).

Nucleotide-binding oligomerization domain-like receptors (NLRs) are intracellular PRRs capable of recognizing a wide array of DAMPs and MAMPs including reactive oxygen species (ROS) (36), organelle calcium efflux (37), Lipopolysaccharide (38), peptidoglycan (39), and viral RNA (40). Prototypically NLRs contain an N terminal effector domain, a central NACHT/nucleotide binding domain, and C terminal leucine rich repeats. NLRs can activate several innate immune pathways following ligand engagement, including the NFκB, MAPK, interferon and inflammasome assembly pathways (36, 39, 41). *H. vulgaris* and two stony coral species have been shown to have large NLR repertoires, containing unique domain combinations that are not seen in mammalian NLRs (10, 42, 43).

C-type lectins (CTLs) are a very diverse protein family that can act as either soluble or transmembrane PRRs (5, 44). They are characterized by the C-type lectin domain (CTLD) which is most well-known for calcium dependent carbohydrate binding but is also capable of binding to proteins, lipids, and inorganic compounds (44). CTLs can activate NFκB as well as the lectin complement pathway (45, 46). A bioinformatic study of *N. vectensis* CTLs also found a large repertoire that could not be categorized by the mammalian CTL classification system (47).

Our study aims to build upon the current base of knowledge on cnidarian PRRs by expanding to investigate four PRR types in a phylogenetically diverse group of cnidarians. Previous studies are heavily concentrated in two classes, the anthozoan class Hexacorillia (stony corals and anemones), and two model systems within the medusozoan class Hydrozoa: *H. vulgaris* and *Hydractinia symbiolongicarpus* (8, 9, 15, 26, 27, 42, 47, 48). To date, PRRs have not been investigated in cnidarians with a free-swimming adult medusae form, meaning that current studies also lack diversity in terms of life history strategies. Furthermore, only one anemone species, *N. vectensis*, has studies of all four PRR types (9, 25, 43, 47). Thus, we lack knowledge of the number and structure of PRRs in the remaining classes of the phylum and the full PRR repertoires of even the well-studied species.

Within the past couple of years, a wealth of cnidarian genomic resources has become available, particularly in the medusozoan clade, making it possible to investigate PRRs in a far more diverse set of cnidarian species (nine anthozoans and six medusozoans), with strong potential to provide a more detailed picture of PRR and innate immune evolution (49–53). Therefore, we surveyed the proteomes of 15 cnidarians, nine anthozoans and six medusozoans, for putative TLRs, RLRs, NLRs, and CTLs with the hypothesis that medusozoans would have less diverse PRR repertoires. Next, because TLRs, RLRs, NLRs, and CTLs are all capable of activating NFκB signaling in mammals, we investigated the PRR to NFκB pathways in all 15 cnidarian species, as well as the lectin complement pathway to determine if there is a disparity in downstream immune pathway completeness between the anthozoan and medusozoan clades.

## Materials and Methods

### PRR Survey

The proteomes of *Acropora millepora* (54), *Actinia tenebrosa* (53), *Aurelia* sp. (50), *Calavadosia cruxmelitensis* (51), *Cassiopea xamachana* (55), *Clytia hemisphaerica* (49), *Dendronephyta gigantea* (56), *Exaiptasia daiphana* (previously *Exaiptaisa pallida*) (57), *Hydra vulgaris* (14), *Montipora capitata* (58), *Morbakka virulenta* (59), *Nematostella vectensis* (60), *Orbicella faveolata* (61), *Pocillopora damicornis* (62), *Xenia* sp. (52), and sponge *Amphimedon queenslandica* (63) were surveyed for TLRs, RLRs, NLRs, and CTLs using HMMR (64). A summary the clade, class, life history traits, genome assembly size, and predicted proteins for each species can be found in **Supplementary Table 1**. All proteomes used were genome based with the exception of *N. vectensis*. This was due to a failure to find the complete prototypical TLR in the genome-based proteome of *N. vectensis*, despite several previous studies reporting its presence in the genome (25). The complete prototypical TLR was also absent in smallest transcriptome shotgun assembly available (NCBI GenBank: HADO000000000.1), so the second smallest transcriptome shotgun assembly (NCBI GenBank : HADN000000000.1) was filtered and used (60). Transdecoder was used to extract the longest open reading frame of each contig and translate it into a predicted peptide sequence (65). Then CD-HIT was used to collapse sequences with a similarity level of 0.85 to limit the number of splice isoforms in the assembly (66). This resulted in a proteome with 42,379 contigs, 14,000 higher than the average number of predicted proteins in the genome-based proteomes used in this study (**Supplementary Table 1**) (67). However, PRR numbers are similar across the genome based proteome of *N. vectensis*, NCBI GenBank: HADO000000000.1, and GenBank : HADN000000000.1 (**Supplementary Table 2**) so excess contigs have minimal impact on results.

Queries were made by using Clustal Omega to make an alignment of all human TLRs, RLRs, NLRs, and CTLs respectively (68, 69). All sequences that had an E-value less than 10^-4.9^ following the HMMR search were then run through Pfam to predict protein domains using the batch search tool (70). Non-repeat domains with an individual E-value of less than 10^-4.9^ were counted and repeat domains were counted if they had an individual E-value less than 10^-3^. TMHMM was used to predict transmembrane domains in TLRs and CTLs (71).

**Figure 1:**
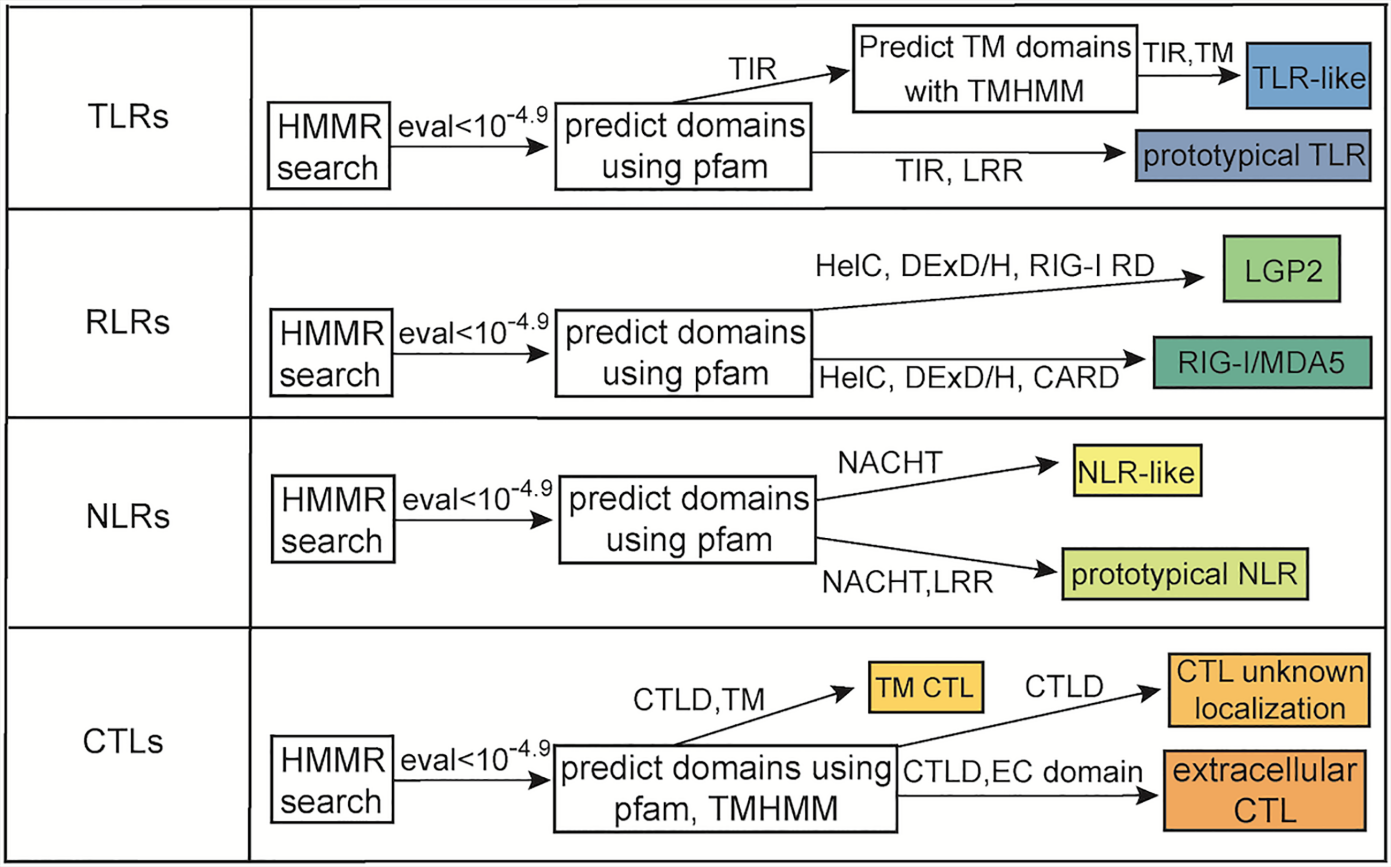
Flow chart of methodology used to identify PRRs. Shown is the minimum domain requirements for a protein to be classified in the various categories as well as homology requirements based upon E-value.

TLRs were classified as prototypical if the predicted protein had both a TIR domain, LRR, and transmembrane domain that met our inclusion threshold. Proteins with a TIR domain and a transmembrane domain were classified as TLR-like proteins. Proteins with a DExD/H box helicase, CARD, and C terminal RIG regulatory domain were classified as RIG-I/MDA5-like receptors. Proteins were classified as LGP2-like receptors if they had a DExD/H box helicase, and RIG-I repressor domain (**Figure 1**). In several proteins the DExD/H box helicase domains had E-values meeting our inclusion threshold but were not considered as a member of the Pfam domain clan following post processing. However, given that Cnidaria is a phylogenetically distant and basal phyla, these domains were counted given the context of the surrounding domains which always included the RIG-I repressor domain. NLRs were classified as prototypical if the predicted protein contained at a minimum both the NACHT domain and LRRs and as NLR-like if they contained at a minimum the NACHT domain. CTLs were divided into 3 groups. Proteins with the CTLD and a transmembrane domain, proteins with CLTD and a Pfam domain indicative of extracellular localization, and proteins with the CTLD and no additional domains indicative of where they may be localized (**Figure 1**). Pfam domains considered to be indicative of extracellular localization included cysteine rich secretory domain (72), CUB (73), F5/8 (74), ShK (75), Von Willebrand factors (76), thrombospondin (77), trefoil (78), Fibrinogen β/γ terminal globular domain (79), NIDO (80), PKD (81), coagulation factor Xa inhibitory domain (82), U-PAR/Ly6 (83), complement Clr like EGF (84), and Xlink (85).

The total number of TLRs, RLRs, NLRs and CTLs in each species were then used as input for ancestral state reconstructions. The phylogenetic tree used for this analysis was created using Orthofinder (86). Ancestral state reconstructions based on maximum likelihood (ML) were made using the fastAnc function in the R package phytools. Phytools was also used to visualize the ML ancestral state reconstructions (87).

### Relationship to Clade and Life History Traits

Principal component analysis was run in R and visualized with the R package ggfortify (88). The input of the PCA analysis was the total number of PRRs in each PRR type per species. To test for associations between total PRR number and clade, intracellular algal symbiosis, coloniality, and mobility, generalized linear models were run in R. Because there is considerable overlap between anthozoan species, symbiotic species, colonial species, and sedentary species, five individual models were tested (total PRRs~trait, family = quasipoisson) (**Supplementary Table 1**).

### Downstream Immune Pathway Completeness

#### NFκB

BLASTp was used to identify NFκB and IKBA in all species included in the study (89). Human sequences of p100, p105, and IKBA were used as queries (69). The top 5 best hits to each query were then run through Pfam to predict domains using the same inclusion thresholds as the PRRs. Because the *N. vectensis* NFκB is known to be truncated only the Rel DNA binding domain and Rel dimerization domain were required for a cnidarian protein to be considered NFκB (90). Multiple ankyrin repeats were required for a cnidarian protein to be considered IKBA.

#### PRR to NFκB Pathway

Cnidarian proteomes were searched for members of the PRR to NFκB KEGG pathways using BLASTp and a human query (69, 89, 91). The cnidarian protein with the highest E-value to each query was then blasted against the human proteome (GCA_000001405) (69). If the human query and best human hit following reciprocal blast were the same protein, the protein was counted as present in the cnidarian.

#### Lectin Complement Pathway

Because complement consists 5 mosaic protein families that likely did not expand until teleosts, we used pBLAST to search for representatives of the C3, Factor B/C2, MASP, and C6 families (89, 92). Human sequences for all members of a given family were used as queries (69). Each protein family has a unique domain composition so the top 5 best hits to each query were run through Pfam using the batch search option to predict domains (70). Proteins were considered members of the C3 family if they contained multiple macroglobulin domains, CUB, and C345c. Proteins were considered members of the Factor B/C2 family if they contained sushi repeats, von Willebrand factors, and a serine protease. Proteins were considered members of the MASP family if they contained 2 CUB domains, sushi repeats, and serine protease. Proteins were considered members of the C6 family if they contained TSP, low density lipoprotein receptor domain class A, MACPF, and sushi repeats (92).

## Results

### PRR Survey

**Figure 2:**
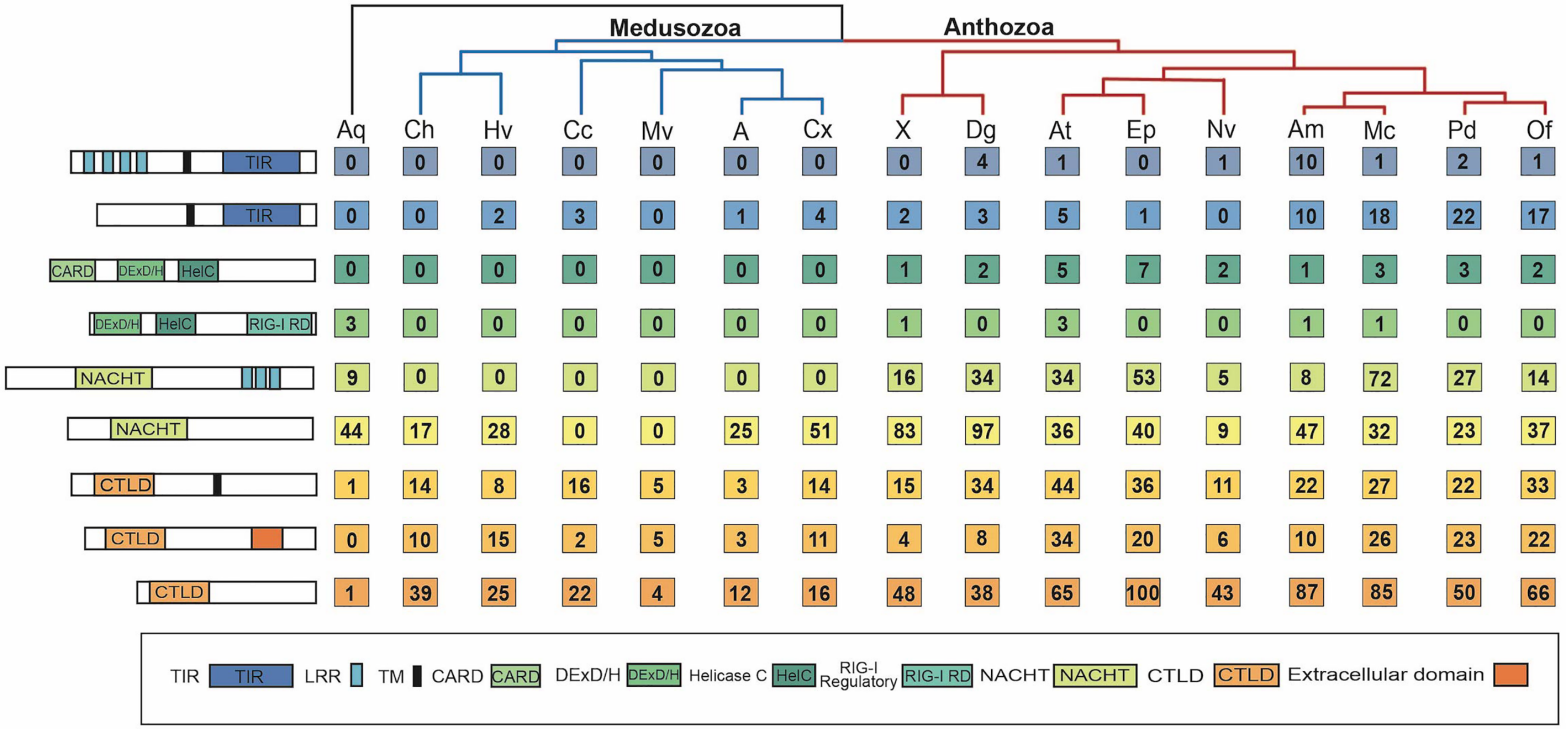
Number of PRRs of each type found in each species. Far left shows schematics of the minimum domain composition for each PRR type, from top to bottom: prototypical TLR, TLR-like, RIG-I/MDA5-like, LGP2-like, prototypical NLR, NLR-like, transmembrane CTL, extracellular CTL, CTL with unknown localization. To the right of the schematics is the corresponding number of each PRR type found in each species’ proteome. Species are grouped by phylogeny, with anthozoans in red, medusozoans in blue, and the sponge outgroup in black. Aq, Amphimedon queenslandica; Ch, Clytia hemisphaerica; Hv, Hydra vulgaris; Cc, Calavadosia cruxmelitensis; Mv, Morbakka virulenta; A, Aurelia sp.; Cx, Cassiopea xamachana; X, Xenia sp.; Dg, Dendonephthya gigantea; At, Actinia tenebrosa; Ep, Exaiptasia daiphana; Nv, Nematostella vectensis; Am, Acropora millepora; Mc, Montipora capitata; Pd, Pocillopora damicornis.

**Figure 3:**
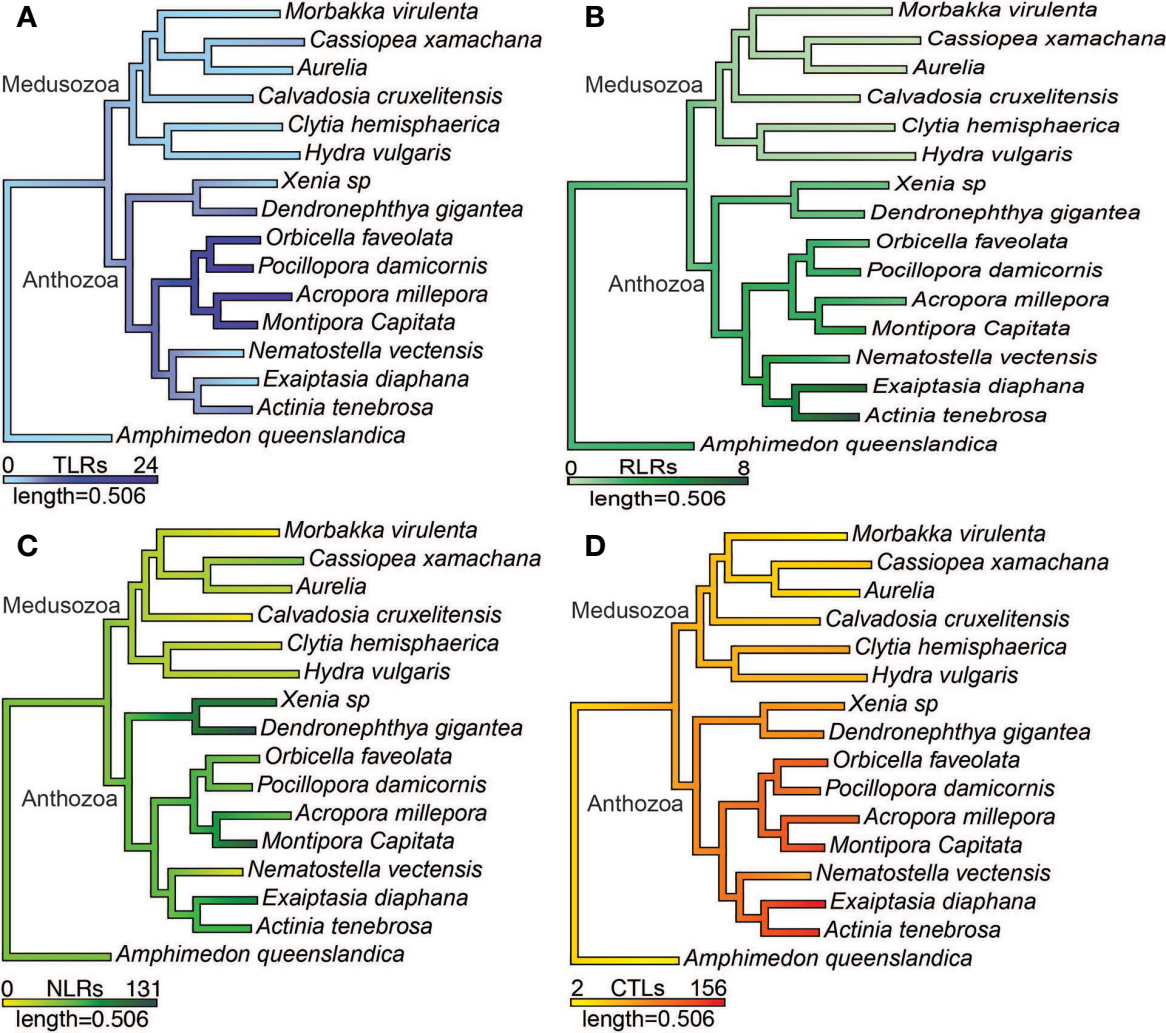
Ancestral state reconstructions of the number of **(A)** prototypical TLRs and TLR-like proteins, **(B)** RIG-I/MDA5-like receptors and LGP2-like receptors **(C)** prototypical NLRs and NLR-like proteins **(D)** CTLs.

The number of prototypical TLRs and TLR-like proteins vary across the species surveyed from 0 to 24. No prototypical TLRs were found in any of the six medusozoan species and in two anthozoans, *E. daiphana* and *Xenia* sp. (**Figure 2**). In contrast, two anthozoans have expansions in prototypical TLRs, *D. gigantea* and *A. millepora*, with four prototypical TLRs found in *D. gigantea* and ten in *A. millepora*. TLR-like proteins with a TIR domain and a transmembrane domain were found in all species except for *C. hemisphaerica* and *M. virulenta* (**Figure 2** and **Supplementary Table 3**). Based upon ancestral state reconstruction, one TLR was present in the common ancestor shared by medusozoans and anthozoans (**Figure 3A**). Relative to this ancestral cnidarian and other anthozoans, stony corals (*A. millepora, M. capitata, P. damicornis, O. faveolata*) have expansions in TLRs, specifically TLR-like proteins (**Figures 2**, **3A**).

RIG-I/MDA5-like receptors were found in the proteomes of all nine anthozoan species (**Figure 2**). These proteins all contained both the C terminal regulatory domain and the RIG-I C terminal repressor domain (**Supplementary Table 4**). Two species of anemone, *A. tenebrosa* and *E. daiphana*, have expansions in RLRs relative to both other anthozoans and the common ancestor shared by medusozoans and anthozoans (**Figure 3B**). These expansions are largely due to the high number of RIG-I/MDA5-like receptors in these species (**Figure 2**). *A. queenslandica*, the sponge outgroup*, Xenia* sp.*, A. tenebrosa, A. millepora*, and *M. capitata* all contained proteins with domains characteristic of LGP2 (**Figure 2**). In *A. millepora* and *M. capitata*, these proteins had an RNA dependent RNA polymerase domain in addition to the canonical LGP2 domain organization (**Supplementary Table 4**). No RLRs were found in any of the six medusozoan species (**Figure 2**).

Diverse arrays of NACHT containing proteins were found in the majority of cnidarian species surveyed. Prototypical NLRs containing NACHT and LRR domains were present in the sponge *A. queenslandica* and all nine anthozoan species but absent in all medusozoan species (**Figure 2**). No NACHT containing proteins were found in two medusozoan species, the Staurozoan *C. cruxmelitensis* and the Cubozoan *M. virulenta* (**Figure 2**). The majority of anthozoan species were found to have expanded NLR repertoires relative to medusozoans. However, only a smaller subset of anthozoan NLR repertoires are expanded relative to the common ancestor shared by medusozoans and anthozoans (**Figure 3C**).

Anthozoan NLR repertoires are not only generally larger (**Figure 3C**), but also more diverse in terms of domain composition (**Supplementary Table 5**). Excluding LRRs 35 different Pfam domains were found in combination with NACHT across the 15 cnidarian species. Several of these domains are associated with immunity, including Dzip3/hRUL138-like HEPN nuclease (93), caspase recruitment domain (CARD) (94), death domain (DD) (95), ZU5 (96), glycosyl transferase (97), NB-ARC (98), WD-40 repeats (99), and Toll/interleukin receptor (TIR) domain (100). Excluding LRRs, the Dzip3/hRUL138- like HEPN domain was the most common domain found in combination with NACHT in *C. hemisphaerica, Xenia* sp.*, D. gigantea, A. tenebrosa, E. daiphana, A. millepora, M. capitata, P. damicornis, and O. faveolata*. Anthozoans show large expansions of proteins with Dzip3/hRUL138- like HEPN domain fused to NACHT, which ranged from 17 proteins in *O. faveolata* to 83 in *D. gigantea*. TIR was the most common domain found with NACHT in *C. xamachana* and DD was most common domain found with NACHT in *H. vulgaris*. Domains associated with transposable elements were found with NACHT in two species, *H. vulgaris* and *M. capitata*. A protein model with NACHT and the hAT family C terminal dimerization domain was found in *H. vulgaris* while *M. capitata’s* proteome contains a protein with NACHT and an endonuclease reverse transcriptase domain and a protein with NACHT and integrase (**Supplementary Table 5**).

All of the cnidarians in this study have expanded repertoires of CTLs relative to the sponge outgroup (**Figures 2**, **3D**). Two medusozoan species that are planktonic as adults, *M. virulenta* and *Aurelia* sp., had the fewest CTLs of the cnidarian species (**Figures 2**, **3D**). Additionally, anthozoans have expanded CTL repertoires relative to both medusozoans and the common ancestor shared by medusozoans and anthozoans (**Figure 3D**). The species with the most predicted CTLs were *A. tenebrosa* and *E. daiphana*, two closely related sea anemones (**Figures 2**, **3D**).

Across all 15 cnidarians, 70 different protein domains were found in combination with CTLD. The two clades differed in the domains most commonly found with CTLD. In six out of the nine anthozoan species the epidermal growth factor domain (EGF) was the most common domain found in conjunction with CTLD. The concanavalin A-like lectin domain was most commonly found with CTLD in four of the six medusozoans and two of the nine anthozoans. In the remaining medusozoans, *C. hemisphaerica* and *M. virulenta*, Von Willebrand factors and cysteine rich secretory domains respectively were most commonly found in conjunction with CTLD. Other common domains fused to CTLD were immunoglobulin, fibronectin, MAM, CUB, cysteine rich scavenger receptor, Kazal-type serine protease inhibitor, and PAN. Proteins with the domain organization of mannose binding lectin (MBL) (CTLD, collagen) were found in *C. xamachana* and *C. hemisphaerica.* As with the NLRs, the CTL search yielded surprising domain combinations. Reverse transcriptase domains were found in combination with CTLD in four species, *M. virulenta*, *H. vulgaris, E. daiphana* and *M. capitata*, and integrase and CTLD were found in *M. capitata*. Additionally, *M. capitata* and *P. damicornis* had predicted proteins with the MAC/Perforin domain in addition to CTLD (**Supplementary Table 6**).

### Relationship to Clade and Life History Traits

**Figure 4:**
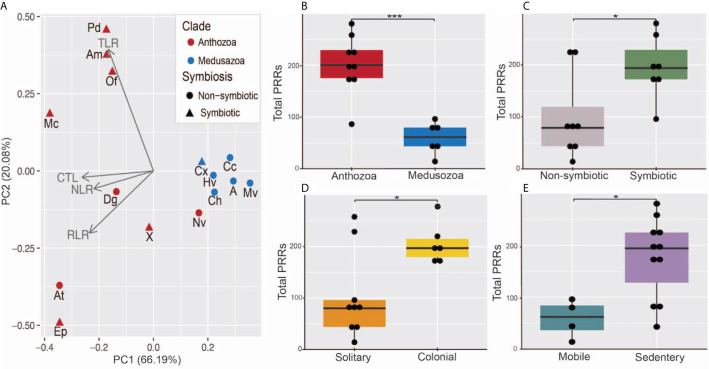
**(A)** Principal component analysis of total number of PRRs in each category, red points indicate anthozoan species, blue points indicate medusozoan species, triangular points indicate symbiotic species, and circular points represent non-symbiotic species. Aq, Amphimedon queenslandica; Ch, Clytia hemisphaerica; Hv, Hydra vulgaris; Cc, Calavadosi cruxmelitensis; Mv, Morbakka virulenta; A, Aurelia sp.; Cx, Cassiopea xamachana; X, Xenia sp.; Dg, Dendonephthya gigantea; At, Actinia tenebrosa; Ep, Exaiptasia daiphana; Nv, Nematostella vectensis; Am, Acropora millepora; Mc, Montipora capitata; Pd, Pocillopora damicornis. **(B–E)** Boxplots of total PRR number separated by **(B)** Clade, **(C)** Symbiotic status, **(D)** Colonial status, and **(E)** motility. *** indicates a p-value less < 0.001 and * indicates a p-value < 0.05 resulting from a generalized linear model. Points represent individual species’ total PRR numbers binned into groups of 10.

Our results indicate the presence of a divide between the two cnidarian clades in PRR number across all four PRR types. Principle component analysis resulted in the medusozoans and a single anthozoan, *N. vectensis*, grouping tightly across both PC1 and PC2. The anthozoans excluding *N. vectensis* group relatively tightly across PC1, driven largely by CTLs and NLRs, but show more variance across PC2. The separation of the anthozoans across PC2 is most likely due to the expansions in prototypical TLRs and TLR-like proteins found in stony corals and the expansion of RIG-I/MDA5-like proteins in *A. tenebrosa* and *E. daiphana* (**Figure 4A**). A generalized linear model found significant associations between the total PRR number and clade (p = 0.0001). Total PRR number was also associated with ability to host intracellular algal symbionts (p=0.038), colonial animals (p = 0.033), and sedentary animals (p=0.014) (**Figures 4B–E**).

### Downstream Immune Pathway Completeness

**Figure 5:**
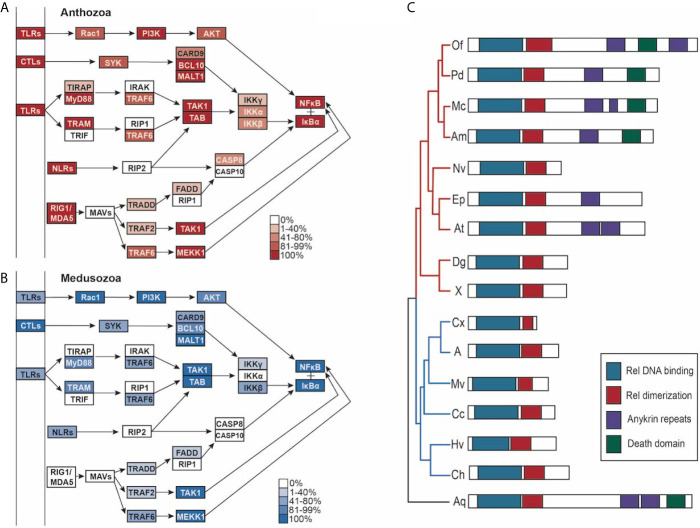
**(A, B)** PRR-NFκB pathways modified from Kegg pathway. Opacity of the boxes represents the percentage of **(A)** Anthozoans or **(B)** Medusozoans with that pathway component. **(C)** schematic of the domains found in NFκB in each species, grouped by phylogeny. Medusozoan species are indicated by the blue phylogenetic tree branches. Anthozoan species indicated by the red phylogenetic tree branches.

Given the differences in PRR numbers across the two cnidarian clades, we then investigated the completeness of the pathways downstream of TLRs, RLRs, NLRs, and CTLs that lead to master immune regulator NFκB (**Figure 5**). All species’ proteomes contained at least one homolog of NFκB. Stony corals and sponge outgroup *A. queenslandica* contain NFκB homologs most similar in domain composition to the mammalian NFκB queries (p100, p105), containing the Rel binding domain, Rel dimerization domain, ankyrin repeats, and the death domain (**Figure 5C**). Anthozoans *E. daiphana* and *A. tenebrosa*, have seemingly lost the death domain in their NFκB, which contains the Rel binding domain, Rel dimerization domain, and ankyrin repeats (**Figure 5C**). Notably, anthozoans *N. vectensis*, *D. gigantea*, *Xenia* sp., and all of the medusozoan species have NFκB homologs that have lost ankyrin repeats and the death domain (**Figure 5C**).

In addition to lacking RIG1/MDA5, prototypical NLRs and prototypical TLRs, medusozoans have slightly less complete PRR to NFκB pathways when compared to anthozoans (**Figures 5A, B**). Notably, IKKγ (NEMO) is absent in the majority of species, although the majority of species. contained at least one IKK (**Supplementary Table 7**). IRAK1/4, TRIF, RIP1, RIP2, MAVs, and CASP10 were missing in all cnidarian species (**Figures 5A, B**). In the case of CASP8, CASP10, and CARD9 the best reciprocal best blast hit was a slightly different caspase or caspase recruitment protein (**Supplementary Table 7**).

**Figure 6:**
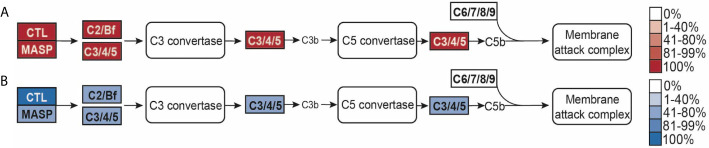
**(A, B)** Lectin-complement pathway modified from Kegg pathway. Opacity of the boxes represents the percentage of **(A)** Anthozoans or **(B)** Medusozoans with that pathway component.

Homologs of MASP, C2, and C3 were found in the majority of species in this study (**Figure 6**). Two medusozoans, *C. cruxmelitensis* and *H. vulgaris* lacked MASP homologs. Notably, all anthozoans had proteins with the domain structure characteristic of the C2/Bf family and C3 family while *C. hemisphaerica* and *H. vulgaris* lacked C2/Bf proteins and *Aurelia* sp. and *C. xamachana* lacked proteins in the C3 family. However, both the *Aurelia* sp. and *C. xamachana* proteomes contained proteins with all of the characteristic C3 family domains except for the C34c domain (**Supplementary Table 8**). No proteins matching the C6-9 protein family’s domain structure were found in any of the species (**Figure 6**).

## Discussion

### PRR Survey

Our study builds upon previous studies of cnidarian PRRs to give a higher resolution picture of PRR evolution within the phylum. We show a clear split in PRR number between two major cnidarian clades, Anthozoa and Medusozoa. Most notably medusozoans lack prototypical TLRs, all RLRs, and prototypical NLRs, while anthozoans possess prototypical PRRs. Further, we show that within Anthozoa extensive expansions of NLRs and CTLs are present in soft corals as well as what has been previously described in stony corals and anemones. In some cases, our results vary slightly from previous studies in the exact number of PRRs identified, due to differences in methodology including queries and search algorithms used, inclusion thresholds, genomic resources, and treatment of splice isoforms (10, 26, 43, 47, 101). These slight differences do not impact the overall findings of this study, including expanded PRR repertoires in the majority of anthozoans relative to medusozoans and a lack of prototypical PRRs in medusozoans not previously reported outside of Hydrozoa (27).

The presence of prototypical TLRs in two anthozoan classes, Octocorallia and Hexacorallia, and the absence of prototypical TLRs in medusozoans, sponges (102, 103), ctenophores (104), and placozoans (105) suggests that within Metazoa prototypical TLRs first appear in the ancestral anthozoan and have been secondarily lost in *Xenia* sp. and *E. daiphana* (57). Anthozoan prototypical TLRs likely are similar in function to the *N. vectensis* TLR which has been shown to play a role in pathogen recognition, NFkB signal transduction, and development (25). Despite their lack of prototypical TLRs, it is likely that *Xenia* sp., *E. daiphana*, and medusozoans are capable of TLR-NFκB signaling through TLR-like proteins, as studies in *H. vulgaris* have indicated (27).

The full mechanism by which transmembrane LRR proteins interact with TLR-like proteins is unknown; however, if multiple transmembrane LRRs are capable of interacting with a single TLR-like protein this pathogen recognition system could provide a versatile source of immune specificity in cnidarians. This study provides further support to previous findings of a lineage specific expansion in TLR-like proteins in stony coral species. This expansion is hypothesized to be involved in distinguishing mutualistic or commensal microorganisms from parasitic microorganisms within the coral holobiont and mounting an appropriate immune response (8, 26, 106). This potential function of TLR-like proteins is particularly vital to understand in corals as microbiome composition is thought to influence resistance to both disease and thermal stress, two existential threats to coral reefs (107, 108)

The presence of the C terminal RIG-I repressor domain indicates that the RIG-I/MDA5-like receptors in anthozoans are more similar to RIG-I than MDA5. This supports previous findings that *N. vectensis* RIG-I/MDA5-like receptors group more closely with vertebrate RIG-I than MDA5 (9). The presence of LGP2-like proteins in *A. queenslandica*, *Xenia* sp., *A. tenebrosa*, *A. millepora*, and *M. capitata* contradicts the hypothesis that RIG-I is the most evolutionarily ancient RLR and supports the hypothesis that LGP2 is the first RLR to emerge (9, 109). The fact that LGP2 is not consistently present in anthozoans indicates either that it is commonly secondarily lost due to low selective pressure or that it has evolved independently several times. Additionally, It is unclear what function LGP2 has in the absence of RIG-I and MDA5; however, the presence of an RNA dependent RNA polymerase domain in the *A. millepora* and *M. capitata* LGP2-like receptors indicates that their LGP2-like receptors most possibly amplify dsRNA targets for RNA interference and do not function like vertebrate LGP2 (34, 35, 110).

The presence of prototypical NLRs in *A. queenslandica* and all nine anthozoan species indicates that prototypical NLRs have been secondarily lost in Medusozoa. However, LRRs appear to not be necessary for DAMP recognition in medusozoan NLRs, as an NLR in *H. vulgaris* was shown to be upregulated in response to both lipopolysaccharide and flagellin (10). Thus, NLR-like proteins may still be a source of immune specificity in cnidarians. As in previous studies, we found expansions of NLRs within anthozoan species, indicating that NLRs are a substantial source of immune specificity within the clade (42, 43). Gene expression studies indicate that NLRs are upregulated in response to immune stressors and modulate apoptosis and immunity (42, 111, 112). Our data support the link between cnidarian NLRs and apoptosis as many of the effector domains found in cnidarian NLRs including CARD (94), ZU5 (96), DD (95), NBARC (98), and WD-40 repeats (99) are associated with apoptotic signal transduction.

Because NLRs are involved not only in pathogen recognition but also in self/altered-self/non-self-recognition, traits such as coloniality and ability to form nutritional algal symbiosis may be linked to expansions in NLRs (18, 113). A recent study indicates that in *E. daiphana*, a symbiotic anemone, microalgae are taken into the cell largely indiscriminately and the decision to retain these microalgae as symbionts or expel them likely occurs intracellularly (114). As intracellular PRRs, NLRs are great candidates for modulating interactions between the algal symbionts and the immune system of their cnidarian hosts during the establishment of symbiosis, a hypothesis with some support from transcriptomic studies (115). 

Currently, knowledge of cnidarian NLRs stems from both bioinformatic and gene expression studies (10, 42, 43, 111, 112) as this family of PRRs has yet to be functionally studied within the phylum. Functional studies of cnidarian NLRs are needed, as our results and previous studies indicate that anthozoans have invested in large and diverse NLR repertoires (42, 43).

The expansion of CTLs across all 15 cnidarian species relative to the sponge outgroup aligns with previous findings of diverse and large CTLD containing protein repertoires in invertebrate species (5, 47). Despite this diversity and the potential for cnidarian CTLs to greatly contribute to immune specificity, functional studies have only been conducted on homologs of mannose binding lectin (MBL), the activator of the lectin complement pathway (11, 116). These studies indicate that MBL homologs in two corals, *P. damicornis* (116) *and A millepora* (11) are capable of binding to both bacteria and Symbiodiniaceae leading to the hypothesis that lectin/glycan interactions are a mechanism of recognition during symbiont infection. This hypothesis has found some support from transcriptomic studies (117, 118). The ability of cnidarian lectins to interact with MASP and activate the lectin-complement pathway has yet to be investigated. Both of the cnidarian lectins for which we have functional studies and the majority of cnidarian species in this study lack CTLs with the collagen helix domain characteristic of MBL (11, 116). However, it is possible that cnidarians are capable of lectin-complement activation despite lacking collagen domains as studies have shown that CTLs lacking collagen domains are still able to interact with MASP (119, 120).

CTLs in cnidarians are potentially a large source of immune specificity which to date has been understudied. Based on their large and diverse CTL repertoires, cnidarians are likely utilizing CTLD containing proteins for a variety of functions. Transcriptomic and proteomic studies indicate that CTLs are involved in coral wound healing (121) and disease response (122, 123) in addition to their hypothesized role in mediating symbiosis with Symbiodiniaceae (11, 116–118). Thus, further functional studies of cnidarian CTLs are warranted and should focus both on highly conserved CTLs and on novel cnidarian CTLs with the potential to shed light on disease processes.

### Relationship to Clade and Life History Traits

There is a divide in number of PRRs and downstream immune pathway completeness between Medusozoa and Anthozoa. There are several life history traits common in the anthozoan clade that could explain a greater need for immune specificity and thus this division (13). Three of these life history traits, intracellular algal symbiosis, coloniality, and sedentary lifestyle, we found to have a significant association with total number of PRRs. While intracellular algal symbiosis occurs in both Medusozoa and Anthozoa, there are far more symbiotic anthozoan genera (18, 124). The establishment and maintenance of intracellular algal symbionts is a complex process, with many cnidarian species hosting several species of algae, and thus it likely requires immune specificity (18). Similar to intracellular algal symbiosis, there are colonial organisms in both Medusozoa and Anthozoa, but they are far more common in Anthozoa. Colonial invertebrates require allorecognition systems to distinguish self-tissues from conspecific tissues, which may necessitate a more diverse repertoire of PRRs (19, 20). Animals that spend the majority of their lifespan motile are far more common in the medusozoan clade. In contrast, anthozoan species are largely sedentary, often residing in microbe rich environments like estuaries and coral reefs (13). As sedentary animals are unable to avoid antigen accumulation through movement, they likely also have a greater need for immune specificity (125).

There are several other life history traits that may be associated with immune specificity in cnidarians that we were unable to test due to either a lack of data or low sample size. Number of mutualistic and commensal bacterial symbioses is almost certainly factor in the amount of immune specificity a given cnidarian has, however this information is not available for many of the species in this study (126). It is also possible that anthozoans and medusozoans have a similar need for immune specificity and simply employ different methods to meet this demand. Medusozoans could rely more heavily on other pattern recognition receptor types, such scavenger receptors, rather than TLRs, RLRs, NLRs, and CTLs to provide their immune specificity (127). Other possible sources of immune specificity not reflected in PRR number include increased substitution rates and post translational modifications (128, 129). With future studies and increased resolution of genomic and proteomic resources we hope that this study can be used as a basis for linking life history to mechanisms of immune specificity.

### Downstream Immune Pathway Completeness

Our study indicates a complex history of NFκB within Cnidaria, as ankyrin repeats appear to have been secondarily lost at least three times within the phylum. This C-terminal inhibitory domain prevents NFκB from trafficking to the nucleus and must be removed *via* proteolysis in order for NFκB to bind to DNA. This regulatory function may not be under strong selective pressure within Cnidaria. There are several functional studies on cnidarian NFκB proteins, including *N. vectensis* Nv-NFκB, which is truncated and does not include ankyrin repeats (90, 130). These studies show that *E. daiphana*’s NFκB binding specificity is more similar to both human NFκB p50 and Nv-NFκB than c-Rel and RelA, despite the Nv-NFκB having a similar domain organization to c-Rel and RelA (130). Because ankyrin repeats appear to have been independently lost, it is unclear if soft coral and medusozoan NFκB share similar binding specificity to Ep-NFκB, Nv-NFκB, and human NFκB p50. However, several studies in both medusozoans and anthozoans show that NFκB is responsive to pathogen exposure (27, 111, 112, 130).

Consistent with previous studies, we found that while cnidarians have the majority of proteins in PRR to NFκB pathways they are missing some components found in mammalian PRR to NFκB pathways (15, 130). However, the absence of these components does not mean that cnidarians are incapable of PRR to NFκB signaling (27, 130). Cnidarians can compensate for missing pathway components either by proteins upstream of the missing component interacting directly with proteins further downstream or through proteins that are not homologous to mammalian pathway members but are able to functionally replace them (10, 131).

While cnidarians likely retain PRR to NFκB signaling through these mechanisms, these missing proteins still indicate potential fundamental differences between cnidarian and mammalian innate immune pathways. Functional replacements may not be regulated or regulate immune pathways in the same manner as their mammalian counterparts. For example, although it appears as though cnidarians have a functional replacement for RIPs, this functional replacement likely is not regulated in the same manner as RIPs because the adaptor proteins RIPs interact with in the decision to promote pro-life NFκB signaling or cell death signaling are also absent (10, 132). The lack of RIPs in cnidarians could result in a greater propensity for cell death signaling over pro-life NFκB signaling, a hypothesis that could explain disease phenotypes in white syndrome coral diseases (133). The absence of MAVs from the RLR-NFκB pathway is notable, as it is unclear how RLR antiviral signaling occurs in the absence of this key adaptor protein, however interactions with other CARD domain containing proteins may mediate RLR signaling in cnidarians (31, 32).

No proteins in the C6 family were found in any of the 15 cnidarian species in this study, consistent with previous findings in *N. vectensis*, *E. daiphana* and reef building corals (57, 92, 118). This indicates that cnidarians are unable to form the membrane attack complex and instead use complement for opsonization through C3 (92). While opsonization of microbes by C3 has not been directly shown in cnidarians, transcriptomic studies in anthozoans show that complement signaling is responsive to bacterial pathogens (112, 118). Scyphozoans *C. xamachana* and *Aurelia* sp. lack the complete domain structure of C3. However, because the domain they are missing, C345c mainly functions in interacting with C6 family proteins, this protein may still be able to opsonize (134).

## Conclusions

As a whole, our data indicate that anthozoans have greater immune specificity than medusozoans, with expansions of NLRs and CTLs providing the majority of this specificity. We hypothesize that a greater immune specificity in anthozoans is needed due to life history traits common within the clade, such as being sedentary, having a colonial body plan, and hosting a complex microbiota that includes intracellular algal symbionts. More broadly, our data indicate that studying cnidarian PRRs can give insight not only into where within Metazoa prototypical PRRs arose but also how basal prototypical PRRs function and the systems by which DAMPS and MAMPs were recognized prior to the emergence of these prototypical PRRs. Further investigations into medusozoan immunity would likely provide a greater understanding of non-prototypical pattern recognition systems. The ecological threat coral diseases pose has led to a wealth of knowledge on anthozoan immune responses (135). Placing these studies in an evolutionary context could give further information as to how basal prototypical PRRs function and more broadly how innate immunity evolved.

## Data Availability Statement

The datasets presented in this study can be found in online repositories. The names of the repository/repositories and accession number(s) can be found in the article/**Supplementary Material**.

## Author Contributions

ME and BD conceived of the project. Data collection and bioinformatic analysis was conducted by ME. ME, BD, and LM wrote the manuscript. All authors contributed to the article and approved the submitted version.

## Funding

Funding was provided by NSF grant no. OCE 1712134 and OCE 1928771 to LM, as well as support from UTA Research Enhancement Program and UTA College of Science for support for ME.

## Conflict of Interest

The authors declare that the research was conducted in the absence of any commercial or financial relationships that could be construed as a potential conflict of interest.

## Supplementary Material

Supplementary Table 1 Life history traits and genome assembly statistics of species included in this study.

Supplementary Table 2 Comparison of N. vectensis proteomes.

Supplementary Table 3 TLR domains and sequence IDs.

Supplementary Table 4 RLR domains and sequence IDs.

Supplementary Table 5 NLR domains and sequence IDs.

Supplementary Table 6 CTL domains and sequence IDs.

Supplementary Table 7 PRR-NFkB pathways sequence IDs.

Supplementary Table 8 Lectin complement pathway domains and sequence IDs.
